# Comparison of Electrochemical Immunosensors and Aptasensors for Detection of Small Organic Molecules in Environment, Food Safety, Clinical and Public Security

**DOI:** 10.3390/bios6010007

**Published:** 2016-02-29

**Authors:** Benoit Piro, Shihui Shi, Steeve Reisberg, Vincent Noël, Guillaume Anquetin

**Affiliations:** University Paris Diderot, Sorbonne Paris Cité, ITODYS, UMR 7086 CNRS, 15 rue J-A de Baïf, Paris 75205, France; shshh2008@hotmail.com (S.S.); steeve.reisberg@univ-paris-diderot.fr (S.R.); vincent.noel@univ-paris-diderot.fr (V.N.); guillaume.anquetin@univ-paris-diderot.fr (G.A.)

**Keywords:** immunosensor, aptasensor, ciprofloxacin, enrofloxacin, ofloxacin, tetracyclines, neomycin, tobramycin, ampicillin, kanamycin, sulfonamides, bisphenol A, cocaine, ochratoxin A, estradiol

## Abstract

We review here the most frequently reported targets among the electrochemical immunosensors and aptasensors: antibiotics, bisphenol A, cocaine, ochratoxin A and estradiol. In each case, the immobilization procedures are described as well as the transduction schemes and the limits of detection. It is shown that limits of detections are generally two to three orders of magnitude lower for immunosensors than for aptasensors, due to the highest affinities of antibodies. No significant progresses have been made to improve these affinities, but transduction schemes were improved instead, which lead to a regular improvement of the limit of detections corresponding to *ca.* five orders of magnitude over these last 10 years. These progresses depend on the target, however.

## 1. Introduction

There is a need to detect, for environment, security or medical purposes, various small organic molecules including antibiotics, drugs, toxins, pollutants, and so on. Although highly sensitive and selective, conventional chromatographic and spectroscopic analytical methods are time-consuming and laborious. Moreover, these techniques require expensive equipment, trained operators and tedious pretreatments. The need for disposable tools for monitoring these molecules encourages the development of simple, efficient, continuous, reliable, cost-effective and field-portable screening methods for analysis of environmental contaminants. Biosensors, and particularly electrochemical ones, appear to be an optimal technology. Since 1962 and the first enzyme-based glucose sensing device, there has been explosive development in biosensors in terms of scientific publications.

The figures of merit for biosensors first depend on the bioprobes which are chosen. Herein we reviewed, among the electrochemical biosensors, the most frequently reported bioprobes (antibodies, aptamers, and peptides, when applicable) for frequently reported targets: antibiotics, bisphenol A, cocaine, ochratoxin A and estradiol. In each case, the immobilization procedure was described, as well as the transduction scheme and the limit of detection. We will compare the results from one probe to another and discuss their efficiency for each target, respectively.

### 1.1. Immunosensors

**Figure 1:**
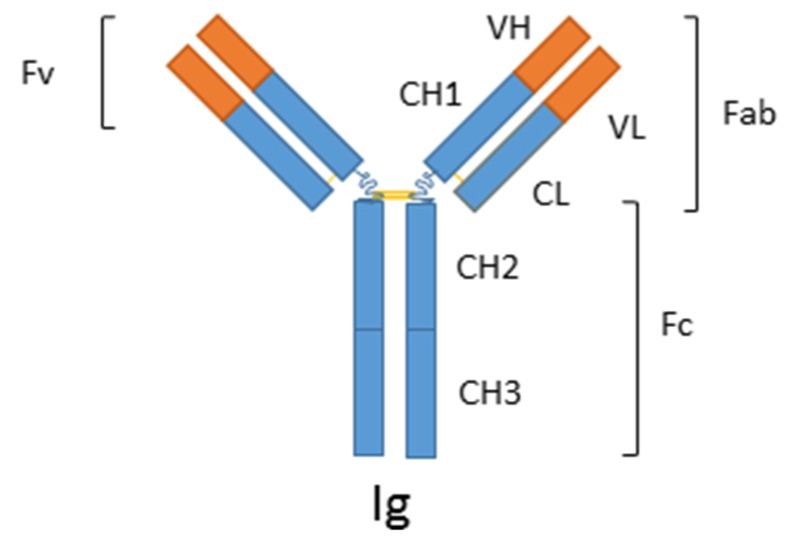
Immunoglobulin G (IgG) consists of two heavy chains (VH, CH1 and CH2) and two light chains (VL and CL). C are constant regions (which do not bring specificity to a given antigen) whereas V are variable regions, which bring specificity. As a consequence, antigens bind to the variable regions VL and VH. In order to decrease the size of IgG, only the Fab (antigen binding fragment, composed of the VH, VL, CH1 and CL regions) can be employed. Reproduced from [3].

Immunosensors are probably the most reported biosensors; they are based on the binding properties of antibodies (Ab) toward a specific target, called an antigen (Ag). The most frequent Ab reported in biosensors are immunoglobulins G (IgG), with a typical molecular weight of 150 kDa and a mean size of 14 × 9 × 4 nm^3^. They are composed of two light and two heavy chains, linked by disulfide bonds to form a characteristic Y-shape [1,2] (Figure 1).

### 1.2. Aptasensors

Aptamers are short (15–100 bases) DNA or RNA strands that are able to bind to a specific target molecule. Aptamers are usually created by selecting them from a large random sequence pool (procedure called SELEX), but naturally occurring aptamers also exist. Aptamers can be used in sensors but were generally developed for clinical purposes, as drugs, for their interactions with expressed proteins. However, they can be selected for other targets than proteins, such as natural or synthetic small organic molecules. Aptamers are helpful in the detection of a wide range of compounds which are not (or too few) immunogenic to allow production of specific antibodies, so that immunosensors cannot be used [4].

### 1.3. Peptide Sensors

The interest in replacing antibodies with peptides is the same as for aptamers [4]. In recent years, the use of peptides as sensing probes for constructing electrochemical biosensors has received great attention because, compared to antibodies or aptamers, peptides are smaller and rarely denaturized when immobilized on the sensing surface. However, even if various peptide sequences exhibiting affinity to particular substrates have been found, peptides capable of recognizing low molecular weight organic compounds with sufficient affinity have rarely been reported as of yet.

## 2. Substances and Receptors

The most frequently reported electrochemical biosensing approaches are comprehensively reviewed for each molecule of interest that we selected.

### 2.1. Antibiotics

**Figure 2:**
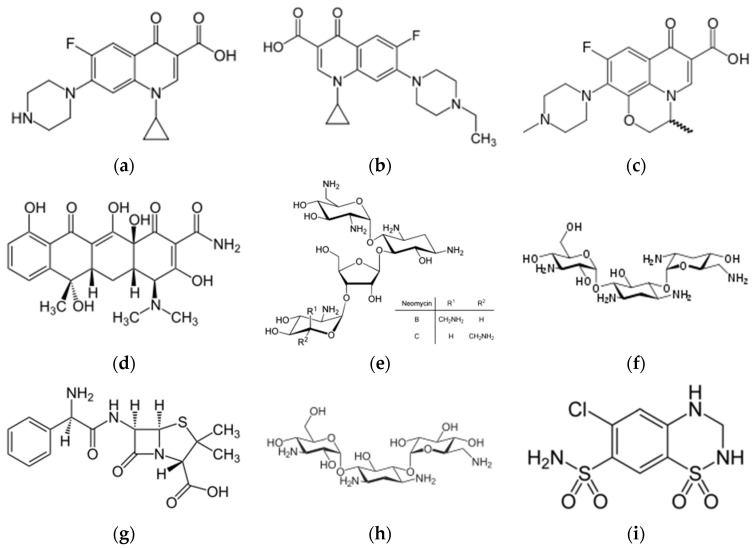
Chemical structures of (**a**) ciprofloxacin; (**b**) enrofloxacin; (**c**) ofloxacin; (**d**) tetracycline; (**e**) neomycin; (**f**) tobramycin; (**g**) ampicillin; (**h**) kanamycin and (**i**) hydrochlorothiazide (a sulfonamide).

Immunosensors for drugs detection and quantification have been described for over 30 years. The most reported drugs are antibiotics and, more particularly, ciprofloxacin, enrofloxacin, ofloxacin, tetracycline, neomycin, tobramycin, ampicillin, kanamycin and sulfonamides (Figure 2). Both antibodies and aptamers were used as probes for these molecules.

#### 2.1.1. Antibodies

**Figure 3:**
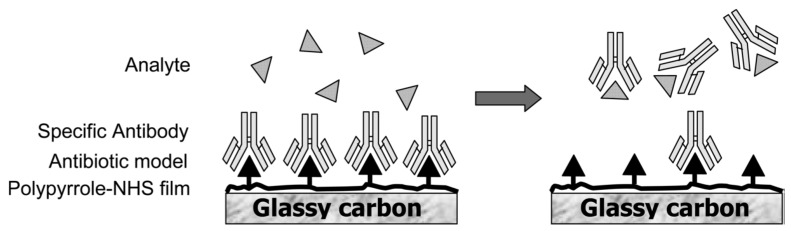
Functioning principle of the immunosensor for ciprofloxacin based on anti-ciprofloxacin antibody displacement. Reprinted with permission from [7]. Copyright 2009 American Chemical Society.

In 2007, Garifallou *et al.* [5] described a label-free immunosensor for ciprofloxacin based on antibody-modified screen-printed carbon electrodes (SPEs), using electrochemical impedance spectroscopy (EIS) for detection. The antibody (Ab) was immobilized through biotin-avidin interactions. The impedance was reported to increase with ciprofloxacin concentration, with a linearity domain between 1 and 100 ng·mL^−1^ (3 nM–0.3 nM). The same year, another group proposed a similar approach, but the Ab was immobilized by peptide coupling, presenting must less steric hindrance than the biotin-avidin coupling, which allowed to decrease the LoD down to 10 pg·mL^−1^ (30 pM) [6]. In 2009, the same group published an innovative immunosensor where ciprofloxacin was first grafted on the electrode, onto which the anti-ciprofloxacin Ab was complexed (Figure 3). In the presence of ciprofloxacin, the antibody was displaced in solution inducing strong changes in the impedance of the electrode; the LoD was of *ca.* 1 pg·mL^−1^ (3 pM) [7].

Detection of enrofloxacin on SAM-modified electrodes gave similar results. For example, Wu *et al.*, (2009) reported EIS characterization of interactions between enrofloxacin and its Ab immobilized on a SAM-modified Au electrode; this interaction caused an increase of electron transfer resistance (Rct) with a LoD of 1 ng·mL^−1^ (3 nM). [8] Zhang *et al.*, (2013) described a competitive enzyme-amplified immunosensor (using the horseradish peroxidase enzyme, HRP) based on gold nanoclusters/polypyrrole (PPy)-functionalized electrodes, for sensitive detection of ofloxacin [9], which exhibited a response in the range 0.08–410 ng·mL^−1^, with a LoD of 30 pg·mL^−1^ (0.1 nM).

The use of magnetic beads (MBs) can improve sensitivity. For example, Zacco *et al.* [10] described an electrochemical immunosensor for the detection of sulfonamide antibiotics based on magnetic beads, amplified with the use of HRP as label. The LoD was *ca.* 1 μg·L^−1^ (6 nM). Centi *et al.* described a more complicated transduction architecture [11], using protein A immobilized on magnetic microbeads (MBs) to bind the specific Ab. A competition assay between the target analyte and the HRP-labeled sulfonamide target was carried out. The LoD was *ca.* 1 ng·mL^−1^ (4 nM). Conzuelo *et al.* (2012) [12] used a selective capture Ab immobilized on the surface of MBs for tetracycline detection. A direct competitive immunoassay using HRP for the enzymatic labeling was performed, with hydroquinone (HQ) as redox mediator. They obtained low LoD (in the ng·mL^−1^ range, *i.e.*, the nM range) for 4 tetracycline antibiotics. The same group described more recently a similar procedure where Abs were immobilized by the diazonium route [13]. Que *et al.* [14] nanostructured their electrodes for tetracycline detection using platinum NPs deposited on graphene nanosheets (GN). This way, retaining a competitive immunoassay format, they significantly lowered the LoD down to 6 pg·mL^−1^ (13 pM).

Even if, as it is generally the case for immunosensors, enzyme-labeling is the dominant strategy for improving sensitivity, it appears that non-enzymatic amplification (through increase of the active surface area or use of catalytic reactions) are often more efficient than classical (HRP) enzymatic amplification.

#### 2.1.2. Aptamers

Aptamers have been used for tetracycline detection as well, by Kim *et al.*, in 2009. They used interdigitated gold electrodes modified with a 76-mer thiol-ssDNA aptamer. The dissociation constant (KD) of this aptamer for oxytetracycline was 11 nM. Transduction was explained by the fact that the redox probe diffusing in solution (Fe(CN)_6_^3−/4−^) was influenced by interactions between the aptamers and the target molecules. The dynamic range was 1–100 nM (LoD of 1 nM, *i.e.*, 0.3 ng·mL^−1^) [15].

In 2010, the same group [16] described similar results but using a streptavidin-modified screen-printed gold electrode onto which biotinylated ssDNA aptamers were immobilized. Still using CV and SWV with Fe(CN)_6_^3−/4−^ as diffusing probe, they found a LoD for tetracycline of 10 nM (*i.e.*, 3 ng·mL^−1^). In another similar example, Zhou *et al.* [17], still using diffusing Fe(CN)_6_^3−/4−^ as redox probe, described an electrochemical tetracycline aptasensor involving multiwalled carbon nanotubes (MWCNTs), the electroactivity of which being decreased due to the formation of aptamer—tetracycline complexes. The peak current changes obtained by differential pulse voltammetry (DPV) increased linearly with tetracycline concentrations from 5 pM (1.5 ng·mL^−1^) to 50 μM. Chen *et al.*, in 2014 [18], used EIS with a 13 bases-long aptamer; a linear relationship between the log concentration of tetracycline and the charge transfer resistance was found from 5 ng·mL^−1^ to 5 μg·mL^−1^; the LoD was 1 ng·mL^−1^ (*ca.* 2 nM). This LoD was significantly lower than the K_D_ determined by the authors using calorimetry (K_D_ of 50 μM). Shen *et al.* [19] used differential pulse voltammetry (DPV) with Prussian blue immobilized onto AuNPs modified with a 79 bases long aptamer having a KD of 63 nM for tetracycline. The detection range was linear from 10^−9^ to 10^−5^ M, with a LoD of 0.3 nM (*i.e.*, 0.1 ng·mL^−1^).

**Figure 4:**
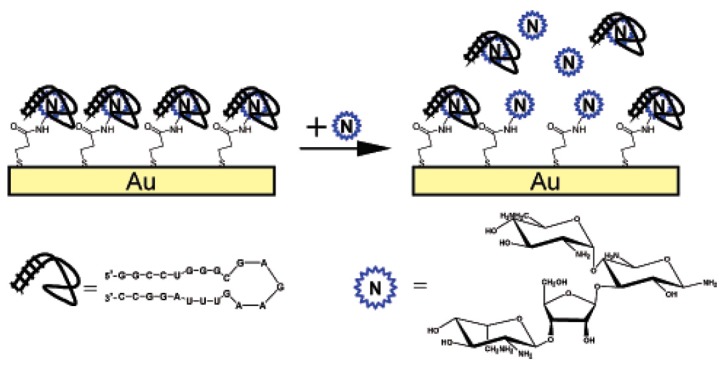
Schematic illustration of the modified electrode and the competitive assay between complexed neomycin on the electrode surface and freely diffusion neomycin. Reprinted with permission from [20]. Copyright 2007 American Chemical Society.

The first label-free electrochemical RNA-based aptasensor for detection of an antibiotic was described in 2007 for neomycin (Figure 4) [20]. The authors described a competitive displacement assay and used EIS. The selectivity of the RNA aptamer allowed determination of neomycin even in biological samples of high protein content. The sensitivity was not excellent, around 1 μM, close to the K_D_ of the aptamers-neomycin complex.

The previous examples are mostly based on the use of a redox probe diffusing in solution, which brings sensitivity but is obviously difficult to integrate when a system is really applied. In 2010, the group of Plaxco showed that classical RNA aptamers, even if sensitive to nuclease, can be used in biological samples providing that these samples were previously ultra-filtrated. They demonstrated the detection of tobramycin in the range 2–6 μM (4–10 μg·mL^−1^) in blood using a methylene blue (MB)-modified RNA aptamer sequence 5′-HS-GGG ACU UGG UUU AGG UAA UGA GUC CC-NH-MB-3′, having a dissociation constant of 13.2 μM for tobramycin (measured in homogenous conditions) [21]. They have shown that the K_D_ increased at least by one order of magnitude in heterogeneous conditions. A similar approach has been reported in several following papers, with variable results. For example, Liu *et al.*, in 2014 [22], reported an electrochemical DNA aptamer-based tobramycin sensor (5′-HS-C6-GGG ACT TGG TTT AGG TAA TGA GTC CC-MB-3′ sequence, immobilized on gold microelectrodes). Despite the electrochemical deposition of dendritic gold nanostructures, they reported a high LoD of *ca.* 1 mM (K_D_ in the mM range as well).

The reagentless detection of small molecules using aptamers is not straightforward because the spatial reorganization triggered by the analyte is often small. To address this issue, Gonzales-Fernandes *et al.* described a strategy based on competition between tobramycin immobilized on magnetic microbeads (MBs) and free tobramycin diffusing in solution. Detection was made by post-labeling aptamers with an enzyme (alkaline phosphatase, AlkP) through the biotin-avidin conjugate. Using a 27-mer anti-tobramycin RNA aptamer (5′-GGC ACG AGG UUU AGC UAC ACU CGU GCC-3′), they found a linear response in the range 5–500 μM [23]. The same authors described later a slightly modified architecture for tobramycin detection using an antibody-antigen association for introducing the enzyme conjugate on the aptamers [24]. Compared with the biotin-avidin approach, the limit of detection was lowered down to 0.1 μM. The authors explained this behavior by the steric hindrance of avidin, which impeded detection.

As shown, reported LoD stay too high for practical applications when aptamers are labeled with redox probes. Another way to detect a molecule through the use of aptamers is to us them as pre-concentrators immobilized on the electrode surface, followed by electro-oxidation or electroreduction of the target molecule directly on the electrode, without any other intermediate. Zhu *et al.* [25] proposed such an approach applied to kanamycin and involving AuNPs. The authors used a 22-bases DNA aptamer (5′-TGG GGG TTG AGG CTA AGC CGA C-3′) showing a high binding affinity to kanamycin (K_D_ = 79 nM), immobilized onto AuNPs modified with poly-[2,5-di-(2-thienyl)-1H-pyrrole-1-(p-benzoic acid)]. Kanamycin detection was achieved by CV and linear sweep voltammetry (LSV). The calibration plot showed a linear range from 0.05 μM to 9.0 μM with a LoD significantly lowered (10 nM, *i.e.*, 5 ng·mL^−1^) compared to the previously reported architectures. Compared to the other amplification strategies used with aptamers, this approach of preconcentration followed by stripping (adsorptive stripping voltammetry—ASV) is certainly the one which leads to the highest sensitivities after EIS.

Lastly, it should also be stressed that attempts to make all-polymer, all-printed, electrochemical sensors were reported. For example, Dapra *et al.* [26] presented an all-polymer electrochemical biosensor based on conductive bilayer consisting of tosylate-doped poly(3,4-ethylenedioxythiophene) (PEDOT-TsO) and its hydroxymethyl derivative which was covalently functionalized with two short aptamer probes with affinity to ampicillin or kanamycin A, respectively. The ampicillin DNA aptamer sequence was 5′-GCG GGC GGT TGT ATA GCG G-3′ (K_D_ = 13 nM) and the kanamycin one was 5′-TGG GGG TTG AGG CTA AGC CGA-3′ (K_D_ = 79 nM). Using EIS, they were able to detect ampicillin in a concentration range from 100 pM to 1 μM and kanamycin A from 10 nM to 1 mM. As discussed by the authors, the low LoD and wide dynamic range of their biosensor could be attributed not only to the high affinity of the DNA probes but also to their device properties. Indeed, for a similar probe applied in different techniques, LoDs can change over three orders of magnitude.

All these results are summarized in Table 1. As shown, tetracycline is a common target for Abs and aptamers. LoDs in the nM range were obtained for both capture probes, without significant differences. For a same probe, it appears that electrode nanostructuration leads to an improvement of the detection limit of at least one order of magnitude. It is also interesting to note that methods involving enzyme amplifications applied with aptamer probes do not appear to be more sensitive than non-amplified methods.

### 2.2. Bisphenol A

**Figure 5:**
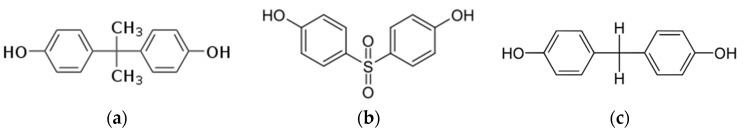
Chemical structure of (**a**) bisphenol A; (**b**) bisphenol S and (**c**) bisphenol F.

Bisphenol-A (BPA) (Figure 5) is a well-known and much studied contaminant causing a wide range of health problems to living beings, especially the young. In 2013, Ragavan *et al.* reviewed the various principles, mechanisms and performances of BPA biosensors available in the literature [27], among which are found electrochemical immuno- and apta-sensors.

#### 2.2.1. Antibodies

The first electrochemical immunosensor for BPA detection was described very recently, in 2007, by Rahman *et al.* and Piao *et al.* (from the same research group) [28,29]. The immunosensor was fabricated by covalent binding of the Ab onto COOH-functionalized polythiophene. It showed specific recognition of BPA on a linear dynamic range between 1 and 100 ng·mL^−1^. The LoD was 0.3 ng·mL^−1^ (1.3 nM). Wang *et al.* published recently a label-free electrochemical competitive immunosensor able to detect BPA directly with a limit of detection of 2 pg·mL^−1^ (9 pM). Characterizations made by SWV showed that the polymer film presented a current decrease upon anti-BPA binding and an opposite current increase upon BPA addition in solution [30]. The difference in sensitivity between the results of Rahman *et al.*, Piao *et al.* and Wang *et al.* could be explained by the nature of transduction scheme. Indeed, the latter reported the use of a competitive immunodisplacement approach whereas the two first articles reported a classical Ab-Ag interaction where the Ab was covalently immobilized on the electrode.

#### 2.2.2. Aptamers

Even if strategies to develop antibodies towards bisphenol A, including antibodies with good affinities, have been reported (see above), DNA aptamers specific to BPA were reported to be of higher affinity than antibodies (in the nM range). For this reason, lower LoDs have been reached. Electrochemical transduction was investigated by Xue *et al.* [31]. They used a specific DNA aptamer against BPA (5′-CCG GTG GGT GGT CAG GTG GGA TAG CGT TCC GCG TAT GGC CCA GCG CAT CAC GGG TTC GCA CCA-3′), immobilized on the surface of a gold electrode via self-assembly and hybridized with its complementary strand. A redox intercalator was added. The detection of BPA was based on the competitive recognition of BPA by the immobilized aptamer on the surface of the electrode, which dehybridized the complementary strand and therefore freed the redox intercalator into solution. The redox signal was recorded by CV. The LoD was 0.3 pg·mL^−1^ (1 pM).

Graphene-modified glassy carbon (GC) electrodes were also investigated, which confers higher specific active areas. Zhou *et al.* [32] immobilized AuNPs on GR-modified electrodes and used freely diffusing Fe(CN)_6_^3−/4−^ as electrochemical probe. The DPV peak current of Fe(CN)_6_^3−/4−^ changed linearly with the concentration of BPA in the range from 10 nM to 10 μM, with a LoD of 5 nM (*ca.* 1 ng·mL^−1^). They used the same aptamer sequence as the previous reference.

#### 2.2.3. Peptides

Specific peptides were also reported for BPA. Yang *et al.* [33] described an electrochemical sensor using the peptide sequence Cys-Lys-Ser-Leu-Glu-Asn-Ser-Tyr-Cys (CKSLENSYC) capable of recognizing BPA with high specificity (isolated using a phage display technique). Peptides were immobilized by thiol adsorption on Au electrodes and BPA was sensed using DPV with Fe(CN)_6_^3−/4−^ as redox probe, with a broad detection range between 1 nM and 5 μM (LoD of 1 nM, *i.e.*, 0.2 ng·mL^−1^). Kim *et al.* also reported very recently the electrochemical detection of BPA with a protein immobilized on RGO electrodes [34]. The protein was a recombinant one obtained by fusing the disulfide-constrained high affinity BPA binding peptide (CKSLENSYC, as reported by Yang *et al.*) to the C-terminus of the Lac repressor (Lad), which is a large protein (140 Å × 60 Å × 45 Å). This modified protein was immobilized on RGO by heat-denaturation, so that it can adsorb by π-stacking interactions. EIS was used (the redox reaction is not given in their manuscript) to probe the presence of BPA down to 100 fM (23 fg·mL^−1^), without interference with bisphenol S and bisphenol F.

All these results are summarized in Table 2. Considering that BPA, Abs, aptamers and peptides have yet been poorly reported in the literature, an unbiased comparison is not possible.

### 2.3. Cocaine

**Figure 6:**
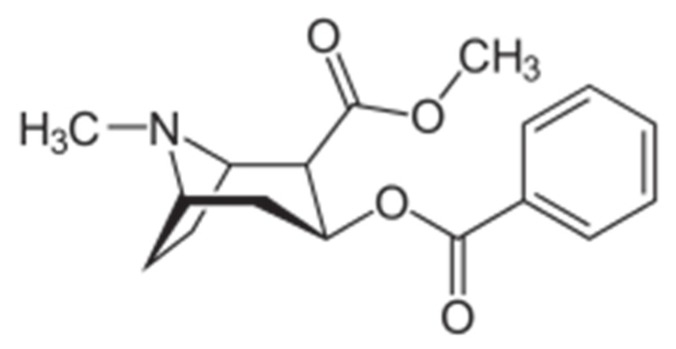
Chemical structure of cocaine, methyl(1R,2R,3S,5S)-3-(benzoyloxy)-8-methyl-8-azabicyclo[3.2.1]octane-2-carboxylate.

Cocaine (Figure 6) is the most frequently used illegal drug after cannabis (*ca.* 15 million users around the world) and directly provokes several thousands of deaths each year. Its detection, e.g., in airports, is routinely made with trained dogs, but more automatic systems would be extremely useful.

#### 2.3.1. Antibodies

There are very few examples of immunosensors dedicated to narcotics. The two very first articles were published in 1998 and dealt with cocaine detection. In 1998, Bauer *et al.* [35] proposed an automated amplified flow immunoassay for cocaine using the alkaline phosphatase (AlkP) enzyme label for amplification and transduction. The LoD was 380 pM. The same year, Suleiman *et al.* [36] described an amperometric immunosensor for cocaine using HRP and, as in the case of Bauer *et al.*, an oxygen electrode. Cocaine was quantified in the range 10^−7^–10^−5^ M, *i.e.*, at too high concentrations for being of practical use.

More than 15 years passed before another example of immunosensor for cocaine or morphine was published. It is only recently that, considering the good results obtained by aptamer-based cocaine sensors, researchers began again to study cocaine-specific antibodies. The literature remains very limited, however. Yang *et al.* [37] described an immunosensor based on a SAM-modified Au electrode for detection of morphine and methamphetamine (MA), using EIS, with a low LoD of 10 pg·L^−1^ (33 fM). For more information, a very recent (2015) book chapter published by Ozkan *et al.*, is available, dealing with electrochemical biosensors for drug analysis [38].

#### 2.3.2. Aptamers

**Figure 7:**
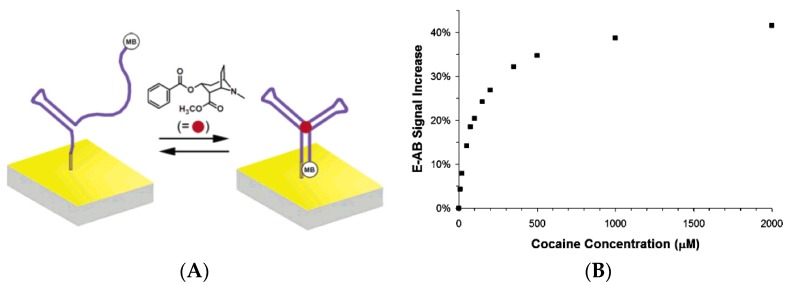
(**A**) Working principle of the aptamer-based cocaine biosensor of Baker *et al.* MB (methylene blue) was used as redox label (**B**) Corresponding calibration curve. Reprinted with permission from [39]. Copyright 2006 American Chemical Society.

By contrast, aptamer-based sensors for cocaine were more frequently reported. The first significant work on this topic was published in 2006 by Baker *et al.* [39]. They demonstrated a rapid, label-free, electrochemical method for the detection of small molecules in general, based on a target-induced conformational change, unaffected by non-specific contaminants, and illustrated by the detection of cocaine in blood serum, saliva, and other complex samples. Two DNA aptamers were investigated (GAC AAG GAA AAT CCT TCA ATG AAG TGG GTC and AGA CAA GGA AAA TCC TTC AAT GAA GTG GGT CG) with K_D_ of approximately 90 μM each, which allowed a LoD below 10 μM (Figure 7); however, these were too high to allow for practical applications.

**Figure 8:**
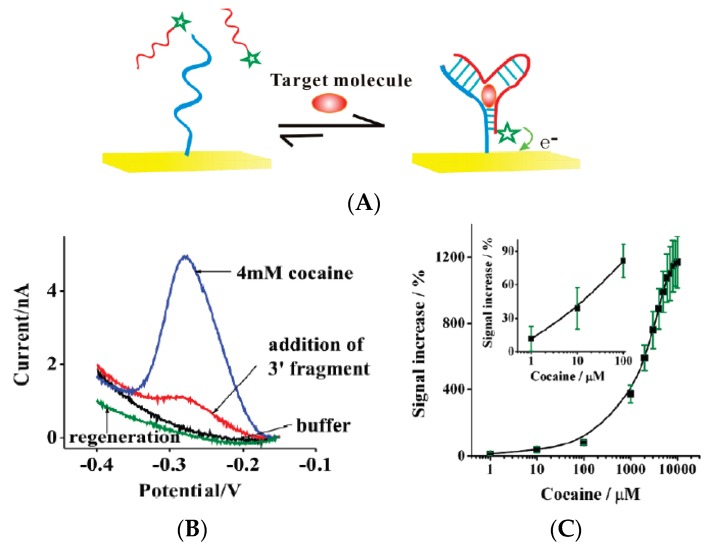
(**A**) Target binding stabilizes the association of the two aptamer strands, leading to a large increase in faradaic current; (**B**) when the target is present; (**C**) Calibration curve. Reprinted with permission from [40]. Copyright 2009 American Chemical Society.

The same group reported later [40] a sandwich assay based on single aptamer sequences labeled with MB and able to detect cocaine. The principle relied on the formation of a complex made of two-strand aptamers which was stabilized together by the presence of the target (Figure 8). Even though the K_D_ was estimated at around 1 mM, the authors reported a significantly lower LoD of *ca.* 1 μM. The limit of quantification (LoQ), however, should be between 0.1 and 1 mM.

**Figure 9:**
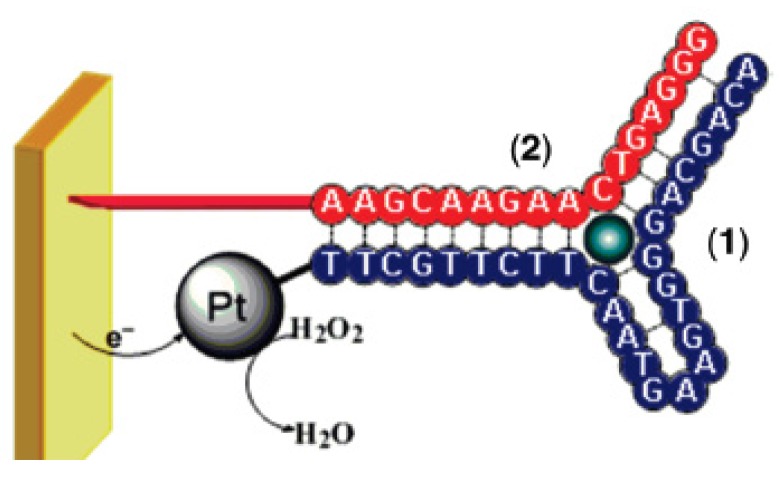
Supramolecular complex of Pt-NPs-aptamer subunit (1) and second aptamer subunit immobilized on the gold surface (2). Adapted with permission from [41]. Copyright 2009 American Chemical Society.

On the same principle, Golub *et al.* [41] described two anti-cocaine aptamer subunits, where one subunit was assembled on an Au electrode, and the second aptamer subunit was labeled with Pt-NPs. The supramolecular Pt-NPs-aptamer subunits-cocaine complex allowed the detection of cocaine by the electrocatalyzed reduction of H_2_O_2_ on the Pt-NPs. The detection limit was in the range of 10^−6^–10^−5^ M (Figure 9).

Again regarding the use of a two-strand aptamers, Du *et al.* [42] constructed a label-free electrochemical aptasensor using layer-by-layer self-assembly of Fc-modified poly(ethyleneimine), with a LoD of 0.1 μM. A similar approach was described by Zhang *et al.* [43] using a primary immobilized aptamer and a secondary aptamer labeled with various quantum dots used in the same way than in bar-code assays, *i.e.*, different QDs yield distinct electrochemical signatures. A LoD of 50 nM was obtained. Another approach using multiple stems was described by Hua *et al.* [44] (with the cocaine DNA aptamer 5′-GGG AGA CAA GGA TAA ATC CTT CAA TGA AGT GGG TCT CCC-3′), where the transduction step was also based on a hairpin structure. The linear range was from 10^−9^ M to 10^−6^ M. Again using the multiple stem approach, Zhang *et al.* [45] investigated the formation of the corresponding supramolecular aptamer fragments/cocaine complex by EIS in the presence of Fe(CN)_6_^3−/4−^. Cocaine concentrations as low as 100 nM were detected. Very recently, Roushani and Shahdost-Fard [46] described a sensor based on the same principle with aptamer-functionalized AuNPs. The interaction of cocaine with the aptamer caused the AuNPs to come close to the electrode surface and impede diffusion of the redox probe Fe(CN)_6_^3−/4−^. Monitored with DPV, the detection limit was 100 pM. The same authors described a similar approach using AgNPs [47] and riboflavin as the redox probe; the LoD was equivalent, *i.e.*, 150 pM.

Jiang *et al.* [48] used the same supramolecular aptamer sequences for detection of cocaine using an alkaline phosphatase-modified binding aptamer, leading to a LoD of 1 nM. A similar approach was used by Zhang *et al.* [49] but using HRP as the amplifying label. DPV was used to obtain a LoD down to 20 nM. Lastly, Shen *et al.* [50] added rolling circle amplification (RCA) in addition to alkaline phosphatase amplification, and used DPV for detection. This dual amplification strategy strongly decreased the detection limit down to 1 nM.

All these results are summarized in Table 3. Due to very few examples of Ab sensors for cocaine, a comparison between Abs and aptamers cannot be objective. As for other targets, nanostructuration of the electrodes led to a gain in terms of LoD, of one to three orders of magnitude. Again, as for antibiotics, enzymatic amplifications applied to aptamers do not bring lower detection limits, as it could be expected. Lastly, even if it is an obvious observation, these results show that the sensitivity largely depends on the affinity of the aptamer for its target: the lowest LoD were obtained with aptamers having low K_D_.

### 2.4. Ochratoxin A

**Figure 10:**
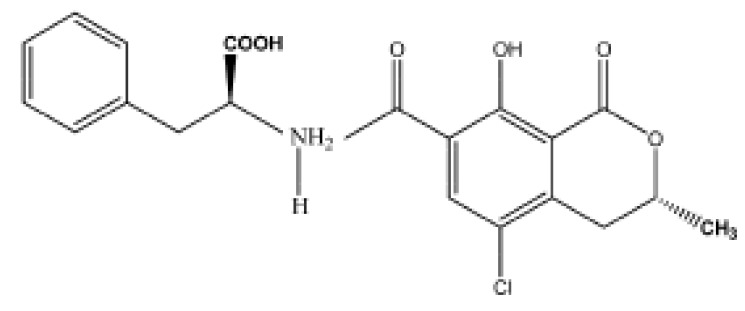
Structure of ochratoxin A (OTA).

A toxin is a poisonous substance produced by living cells or organisms. Toxins can be small organic molecules, peptides, or proteins. The most studied toxin in electrochemical biosensors is ochratoxin A (Figure 10). It is one of the most abundant food-contaminating mycotoxins.

#### 2.4.1. Antibodies

Prieto-Simon *et al.* described, in 2008 [51], two indirect electrochemical competitive enzyme-linked immunosorbent assays (ELISA) for ochratoxin A (OTA). Performances of polyclonal (pAb) and monoclonal (mAb) antibodies against OTA were compared, showing at least one order of magnitude higher affinity values when working with mAb. Alkaline phosphatase (AlkP) and horseradish peroxidase (HRP)-labeled secondary antibodies were evaluated. Similar limits of detection of 0.7 and 0.3 ng·mL^−1^ (0.8 nM) were obtained for HRP- and AlkP-labeled immunosensors, respectively. Later, in 2009, Radi *et al.* [52] described an electrochemical immunosensor for detection of OTA on screen-printed gold electrodes modified by electroreduction of 4-nitrophenyl diazonium salts and anti-OTA. A competition between OTA and HRP-labeled OTA (OTA-HRP) for the immobilized antibodies was held. The activity of the bound OTA-HRP was electrochemically measured by chronoamperometry using 3,3,5,5-tetramethylbenzidine (TMB) as substrate. A LoD of 12 ng·mL^−1^ (30 nM) was obtained. The same authors [53] also investigated a similar architecture using EIS, with a LoD of 0.5 ng·mL^−1^ (1.3 nM).

Zamfir *et al.* described a label-free immunosensor based on magnetic nanoparticles for ochratoxin A [54]. The OTA antibodies were attached to these magnetic nanoparticles (MBs) and afterwards immobilized on a gold electrode pretreated with bovine serum albumin (BSA) under a magnetic field. The impedance variations due to the specific antibody–OTA interactions were correlated with the OTA concentration in the samples. The increase in electron transfer resistance was proportional to the concentration of OTA on a linear range between 0.01 and 5 ng·mL^−1^ (25 pM–13 nM).

An indirect competitive enzyme-linked immunosorbent assay format was also constructed by immobilizing ochratoxin A on gold electrodes [55]. Electrochemical detection was performed by chronoamperometry using TMB and hydrogen peroxide with HRP as label. A LoD of 0.05 μg·L^−1^ (0.12 nM) was achieved.

Additionally, boron doped diamond (BDD) was employed [56] for sensitive immunodetection of ochratoxin A using EIS. Abs were grafted through diazonium functionalization. The increase in electron-transfer resistance presented a sigmoidal shape *versus* log concentration of OTA, with a dynamic range between 7 pg·mL^−1^ and 25 ng·mL^−1^. A LoD of 7 pg·mL^−1^ (17 pM) was obtained. These good results could be attributed to the low background and capacitive currents allowed by BDD compared to other more classical electrode materials.

Lastly, a label-free photoelectrochemical sensor was recently reported by Yang *et al.* [57], by assembly of CdSe nanoparticles on TiO_2_ electrodes. Ascorbic acid (AA) was used as an electron donor for scavenging photogenerated holes under visible-light irradiation. The photocurrent response of the CdSe NPs-modified electrode was significantly enhanced as a result of the band alignment of CdSe and TiO_2_ in electrolyte. OTA antibodies were immobilized on CdSe. Even if these values cannot strictly be compared to the previous ones because the transduction mechanism is not of the same nature, one may underline the good sensitivity of this technique, with a LoD of 2 pg·mL^−1^ (5 pM) and a linear dependence with OTA concentration from 10 pg·mL^−1^ to 50 ng·mL^−1^.

#### 2.4.2. Aptamers

Aptamers were also frequently reported in the literature, for ochratoxin A detection. A first electrochemical platform was described in 2010 using the DNA sequence 5′-GAT CGG GTG TGG GTG GCG TAA AGG GAG CAT CGG ACA-3′ as aptamer probe [58]. In this article, the aptamer was immobilized on the surface of a glassy carbon electrode treated with PCl_5_-activated sulfonic acid groups able to bind amino-modified DNA strands. These primary strands were then sandwiched with two other DNA strands carrying methylene blue (MB) as redox label. Binding of the OTA target to the aptamer disassembled the DNA strands and lowered the MB redox current. The low LoD (30 pg·mL^−1^, *i.e.*, 80 pM) could be attributed to the functionalization strategy (direct coupling of the DNA strands on the electrode without any intermediate bulky groups). One year later, a Langmuir–Blodgett (polyaniline (PANI)–stearic acid (SA)) impedimetric OTA aptasensor was developed by Prabhakar *et al.* [59]. The DNA aptamer was covalently immobilized onto the LB monolayer deposited onto ITO plates. The dissociation constant (K_D_) was found to be *ca.* 0.8 × 10^−7^ M and the LoD was 0.1 ng·mL^−1^ (0.25 nM), *i.e.*, higher than with the direct coupling strategy of Kuang *et al.*

Tong *et al.*, in 2011 [60], described an original signal-on aptasensor based on exonuclease-catalyzed target recycling. To construct the aptasensor, a Fc-labeled probe DNA (5′-Fc-AAA GAT CGG GTG TGG GTG GCG TAA AGG GAG CAT CGG ACA-3′-SH), carrying the Fc label at its 5′-distal end was immobilized on Au electrode through 3′-thiol chemisorption, and hybridized with the complementary ochratoxin A aptamer. In the presence of ochratoxin A, formation of the complex resulted in the transformation of the probe DNA into a hairpin structure where the Fc group comes close to the electrode surface, therefore producing a current increase (signal-on). OTA was then decomplexed by the use of the exonuclease, which digest the aptamer, so that OTA is recycled and can come again to bind to another aptamer. Due to the amplification step, this original method gave a LoD of 1 pg·mL^−1^ (2.5 pM). Zhang *et al.* [61] described a sensor based on a similar hairpin-shaped aptamer and site-specific DNA cleavage of restriction endonuclease TaqaI, with HRP instead of Fc. In their approach, the endonuclease is able to cut DNA (and release HRP in solution, far from the electrode surface) when OTA is absent; if OTA is present, there is no hairpin structure, and, therefore, the TaqI is inactive and HRP can be used as enzymatic amplification label. Due to this amplification, the reported LoD was 0.4 pg·mL^−1^ (1 pM).

Lastly, Hayat *et al.* reported, in 2013 [62], a strategy for the fabrication of an electrochemical label-free (but needing a redox molecule diffusing in solution) aptasensor where long spacer chains of polyethyleneglycol were used to create diffusion channels for the redox probe, while aptamers acted as gate of the tunnels, depending on their conformation. The LoD was 0.12 ng·L^−1^ (0.3 pM). The aptamer sequence was the same as Tong *et al.*

#### 2.4.3. Peptides

Even though it has not yet been applied to electrochemical sensors, peptides were also described in a few works as a probe for ochratoxin A. Indeed, Bazin *et al.* [63] reported in 2013 the peptide NF04 (12-mer sequence: N′-Lys-Cys-Cys-Lys-Tyr-Tyr-Lys-Arg-Asn-Met-Tyr-Val-C′) for specific binding to ochratoxin A, which was evaluated using a peptide-based ELISA assay. Heurich *et al.*, described a computational approach to design peptide ligands for Ochratoxin A [64]. Two peptides were identified: N′-Cys-Ser-Ile-Val-Glu-Asp-Gly-Lys-C′ (octapeptide) and N'-Gly-Pro-Ala-Gly-Ile-Asp-Gly-Pro-Ala-Gly-Ile-Arg-Cys-C' (13-mer). SPR analysis confirmed that the peptides bind to ochratoxin A with KD values of 10 to 15 μM. More recently, Solerai *et al.* [65] reported another peptide conjugated chitosan foam for detection of OTA using enzymatic chemiluminescence. There is therefore no reason for electrochemical OTA sensors based on peptide probes not to appear in the literature in the forthcoming years.

All these results are summarized in Table 4. It appears that OTA sensors are generally more sensitive than for other targets, in the pM range, for both immunologic and aptamer approaches.

### 2.5. Estradiol

#### 2.5.1. Antibodies

**Figure 11:**
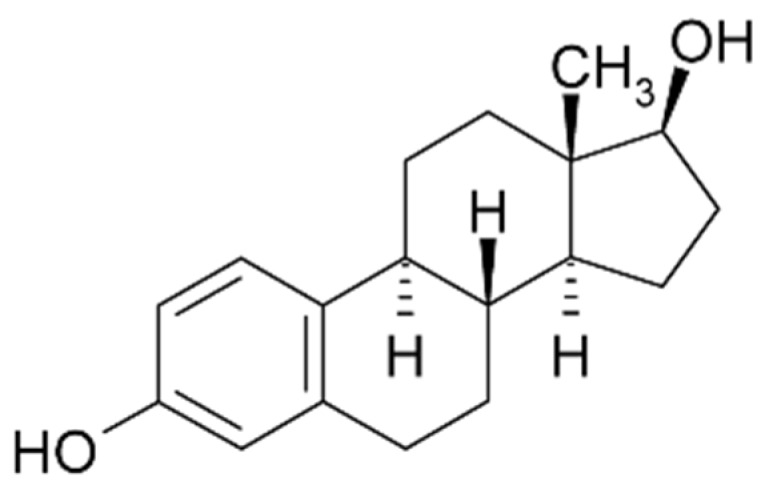
Structure of estradiol (E2).

Estradiol (E2, Figure 11) immunosensors were extensively reported in the literature. In 1998, Padeste *et al.* [66] reported an amperometric immunosensor for estradiol using microperoxidase MP-11 antibody conjugates for amplification. However, in this preliminary work, the authors provided neither LoD nor sensitivity. Five year later, in 2003, Kuramitz *et al.* [67] described a 17β-estradiol-modified electrode for non-labeled immunoassay. When the antibody was bound to the estradiol self-assembled monolayer on the gold electrode surface, they observed a decrease of the electroactivity of the redox probes added in solution (BQ or Fe(CN)_6_^3−/4−^), attributed to the steric hindrance between the antibody on the electrode surface and the redox marker. The authors detected 17β-estradiol, 17β-estradiol-6-one (1,3,5-estratriene-3,17β-diol-6-one 6-O-carboxymethyloxime) and diethylstilbestrol using competition for the antibody between the analyte in solution and 17β-estradiol immobilized on the electrode surface, and found that the affinities ranked as follow: 17β-estradiol-6-one > 17β-estradiol > DES. The LoD for 17β-estradiol was *ca.* 0.1 nM (the KD was estimated around 10^−7^ M).

In 2005, Pemberton *et al.* [68] investigated an electrochemical immunosensor for estradiol on carbon SPE electrodes. A competitive immunoassay was performed using an AlkP-labeled estradiol conjugate. Electrochemical measurements were performed using differential pulse voltammetry (DPV) following the production of 1-naphthol from 1-naphthyl phosphate. The LoD was of 50 pg·mL^−1^ (0.2 nM). In 2006, Butler *et al.* [69] also reported very similar work. The sensor detected 17-beta-estradiol in an environmentally relevant range with a LoD of 0.25 pg·mL^−1^ (0.9 pM). The same year, Volpe *et al.* [70] reported a similar work with a LoD of 40 pg·mL^−1^ (0.15 nM) in bovine serum.

As for the other analytes, nanostructuration of the electrodes improved sensitivity and LoD. For example, Liu *et al.* (2009) [71] reported the use of AuNPs immobilized on gold electrodes in order to increase the surface area and therefore to improve sensitivity. For amplification, they used a HRP-labeled 17 beta-estradiol conjugate and benzoquinone (BQ) as redox reporter. They reported a LoD of 6 pg·mL^−1^ (20 pM). In 2010, the same authors compared SWV and EIS on a SAM-modified Au electrode carrying AuNPs vertical bar thiolated protein G-scaffold to facilitate the immobilization of an enhanced quantity of an almost uprightly aligned anti-estradiol capture antibody [72]. They used Fe(CN)_6_^3−/4−^ as redox probe. They obtained a LoD of 18 pg·mL^−1^ (65 pM) for SWV, and 26 pg·mL^−1^ for EIS (95 pM), e.g., not better than for their previous work. Again using nano/microstructuration, Martinez *et al.* [73] developed a competitive direct immunoassay using magnetic microspheres and an estradiol-HRP conjugate. The detection of estradiol was carried out using a competitive direct immunoassay format using anti-estradiol polyclonal Abs immobilized on 3-aminopropyl-modified magnetic microspheres. Estradiol present in the sample competed with an estradiol-HRP conjugate for the immobilized antibody. The current obtained from the enzymatic reaction was inversely proportional to the amount of estradiol in the sample. The LoD was 0.32 ng L-1 (*ca.* 1 pM).

More recently, in 2012, Kim *et al.* [74] described an impedimetric sensor by immobilizing a monolayer of estrogen receptor-alpha on Au electrodes. The binding of 17β-estradiol increased the electron-transfer resistance of the electrode which was directly monitored by EIS in the presence of Fe(CN)_6_^3−/4−^. Despite the small size of estradiol compared to that of the immobilized antibody, the authors reported a K_D_ of *ca.* 5 pM and a LoD of *ca.* 0.1 pM. Liu *et al.* [75] published an original copper monolayer-based sensor to construct a Cu/vertical bar protein G immunosensor for 17 beta-estradiol. Copper minimized the non-specific adsorption of biological molecules on the immunosensor surface and enhanced the binding efficiency between immunosensor surface and protein G. SWV was employed to monitor the electrochemical reduction current of ferrocenemethanol; SWV current decreased with the increase of estradiol-BSA conjugate concentration. The LoD was 12 pg·mL^−1^ (45 pM).

**Figure 12:**
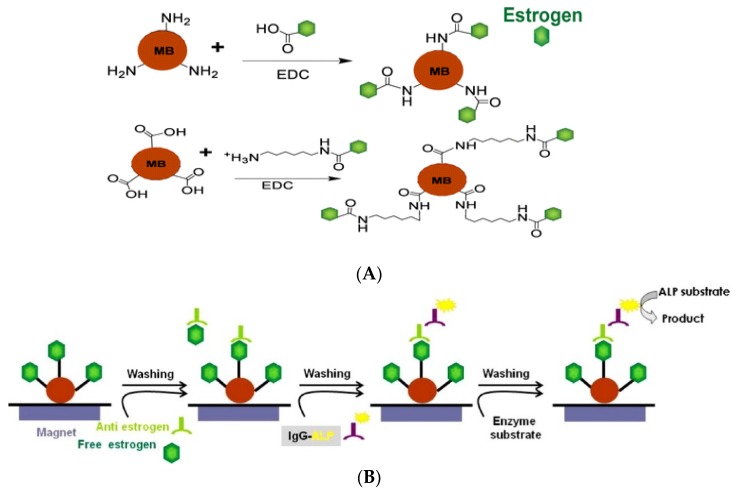
Strategy for (**A**) estrogen functionalization with magnetic beads; (**B**) indirect competitive assay for both colorimetric and electrochemical detection based on functionalized MBs. Reproduced from [77] with permission.

In 2012, Ojeda *et al.*, described a more classical approach [76] based on the surface modification of a carbon SPE with grafted p-aminobenzoic acid (PABA), followed by covalent binding of streptavidin and immobilization of biotinylated anti-estradiol antibodies labeled with HRP. Hydroquinone was used as redox mediator. A LoD of 0.77 pg·mL^−1^ (2.8 pM) was achieved. Kanso *et al.* [77] described two methods using MBs onto which synthetic estrogen derivatives were bound. Using a classical primary antibody and a HRP-labeled secondary antibody, SWV was used for detection of the enzyme product, with LoDs between 1 and 10 ng·L^−1^ (3.6–36 pM) (Figure 12).

Chaisuwan *et al.* described also an original procedure [78] using estradiol-modified CdSe quantum dots (QDs). A bismuth-coated electrode was used for detecting the cadmium ions (Cd^2+^) released during the acid dissolution of the QDs, after the recognition step that occurred in solution. The LoD was *ca.* 52 pg·mL^−1^ (0.2 nM).

Still using nanoparticles, Li *et al.* [79] described an estradiol immunosensor based on graphene-polyaniline (GR-PANI) composites and graphene oxide (GO). HRP-GO-antibody conjugates were used to improve sensitivity, through a competitive immunoassay. The LoD was of 0.02 ng·mL^−1^ (75 pM). Very recently (2015), again using nanostructures, Zhang *et al.* [80] reported a sensor using Cu_2_S nanostructures as labels. Cu_2_S NPs were covalently conjugated to BSA-estradiol. Even without using enzyme-label and acid dissolution, the Cu_2_S generated an intense electrochemical SWV signal giving a LoD of 7.5 pg·mL^−1^ (28 pM). The same authors [81] described a graphene-modified electrode modified with mesoporous Fe_3_O_4_ and loaded with Pb^2+^ or Cd^2+^ cations and estradiol antibodies. In this case, square wave adsorptive stripping voltammetry (SWASV) was used, in order to detect Pb^2+^ or Cd^2+^. SWASV peak currents were proportional to the concentrations of estradiol down to a LoD of 0.015 pg·mL^−1^ (55 fM). Lastly, Jimena *et al.* [82] developed an immunosensor by immobilization of the anti-17 beta-estradiol monoclonal antibody on an Au disk modified with AuNPs on a cysteamine SAM. The limit of detection was 0.84 pg·mL^−1^ (3 pM). To conclude this section and as already remarked in Section 2.1 concerning antibiotics, the most pertinent strategy to reach the lowest limits of detection is to use adsorptive stripping methods [78,81].

#### 2.5.2. Aptamers

Comparatively, aptamer-based estradiol sensors were less reported than their immunosensor counterparts and their use appeared more recently. Olowu *et al.* [83] described in 2010 an electrochemical DNA aptasensor for 17-beta-estradiol based on poly(3,4-ethylenedioxythiophene) (PEDOT) doped with AuNPs. Streptavidin was covalently attached to the electrode and the aptamer immobilized via streptavidin-biotin interaction. The electrochemical signal generated from the aptamer-target molecule interaction was monitored by standard CV and SWV using Fe(CN)_6_^3−/4−^ as redox probe. The signal observed showed a current decrease due to steric hindrance from the bound 17 beta-estradiol. The LoD was 0.1 nM. In 2012, Lin *et al.* [84] reported also a conventional transduction approach based on EIS with Au electrodes modified by a thiolated aptamer. Upon formation of the estradiol/aptamer complex on the electrode surface, the interfacial electron transfer resistance (Ret) increased, with a detection limit of 2 pM. This Ret increased was explained by the fact that the negatively-charged aptamer probe hindered the electron transfer reaction of the redox probe Fe(CN)_6_^3−/4−^ on the Au electrode surface.

More recently, in 2014, Huang *et al.* [85] described a more original nanostructured sensor using flower-like vanadium disulfide, showing ordered nanosheets of several nanometers of thickness onto which the aptamers were immobilized. Very classically, Fe(CN)_6_^3−/4−^ was used as a diffusing redox probe and DPV was used to sense change in apparent diffusion coefficient when 17-beta-estradiol is associated with the grafted aptamers. A detection limit of *ca.* 1 pM was reported. Again exploiting nanostructuration, Ke *et al.* [86] reported a dendritic gold microstructure immobilized on boron doped diamond (BDD) electrode. Estradiol aptamers were immobilized on the surface of the dendritic Au/BDD electrode through Au-S interaction. Once estradiol reacts with aptamers, it enlarges the interfacial electron transfer resistance (sensed by EIS, again using the redox probe Fe(CN)_6_^3−^/Fe(CN)_6_^4−^) on the electrode surface thus cause the increase of impedance) down to a LoD of 5 fM of estradiol. In 2015, Zhu *et al.* [87] reported a nanoporous conducting polymer electrode whose surface potential, modified upon interaction between estradiol and its aptamer, was still probed via EIS using Fe(CN)_6_^3−/4−^ as redox probe. Transduction was explained by the redistribution of negative charges in the electrode double-layer region when the aptamer adopts a folded conformation around the small neutral target molecule. The LoD was in the fM range. Additionally, in 2015, Fan *et al.* [88] used nickel hexacyanoferrate NPs as signaling probe, immobilized on the electrode. AuNPs were put on the NiFe(CN)_6_ NPs, and the thiolated aptamer was immobilized. Upon formation of estradiol-aptamer complex, the interfacial electron transfer reaction (still probed via EIS using Fe(CN)_6_^3−/4−^) of the probe was lowered, resulting in the decrease of the electrochemical signal. The LoD was *ca.* 1 pM.

At last, Jimenez *et al.* [89] described the selection of aptamers having dissociation constants for estradiol in the low nM range (17 nM for the best one). They described an EIS aptasensor based on the conformational change of the aptamer immobilized on a gold electrode by self-assembly. It was shown that the signal of the Fe(CN)_6_^3−/4−^ redox probe was optimized upon hybridization of the aptamer with a short complementary sequence at some specific sites, which was attributed to the more significant conformational change of the aptamer/DNA duplex than the single-stranded aptamer upon binding with the target. The LoD was 0.90 ng·mL^−1^ (3 nM).

All these results are summarized in Table 5. LoDs appear to be lower for estradiol than for the other targets dealt with in this review. Conversely to pollutants or other toxic small organic molecules, antibodies are available and lead to low LoDs, even if aptamers allow to obtain the lower ones (for similar transduction methods). If we compared LoD for a same probe, similarly to the others targets discussed above, the enzyme-amplified strategy applied with aptamer probes do not lead to significant improvements; this a obviously not the case for antibodies, for which enzyme-labelling has long proven its efficiency. Concerning the sensing strategies used with aptamers, EIS coupled to the Fe(CN)_6_^3−/4−^ redox probe is dominant. Efforts could be made to improve this step.

## 3. Conclusions and Perspectives

Electrochemical immunosensors and aptasensors for organic molecules have been significantly reported in the literature since the mid-2000s, whereas peptide sensors remain confidential at this time. Limits of detections are generally two to three orders of magnitude lower for immunosensors than for aptasensors due to the highest affinities of antibodies. No significant progresses have been made to improve these affinities, but transduction schemes were improved instead, which led to a regular LoD improvement corresponding to *ca.* five orders of magnitude over these last 10 years.

If we look more closely to each target molecule discussed in this review, one sees that no significant progresses was made for antibiotics, for which immunosensors are still the most efficient compared to aptasensors, with an average LoD of *ca.* 10^−10^ M *versus* 10^−8^ M, respectively. There are very few immunosensors for cocaine, whereas aptasensors, which appeared in 2006, are frequently reported and show an improvement of their LoD of five order of magnitudes over the last 10 years. Concerning ochratoxin A, there is no difference between immuno- and apta-sensors in terms of limit of detection, which progressed by two to three orders of magnitude over the last 10 years. Lastly, concerning estradiol, there is no significant difference between immuno- and apta-sensors in terms of limit of detection, and very little improvement has been made since the first publications.

From the existing literature, we did not identify obvious parameters which could be used to predict affinities of captures probes (antibodies or aptamers) for a given target. As stressed above, generally speaking, antibodies still present higher affinity constants for their targets than aptamers. This not only leads to lower LoD, but also to a better specificity when targets are sensed in complex matrixes. However, aptamers are more pertinent than antibodies when enantioselectivity is necessary, which is particularly pertinent for drug analysis.

## Figures and Tables

**Table 1:** Figures of merit of selected immunosensors and aptasensors for detection of some antibiotics: ciprofloxacin (CF), ofloxacin (OFL), sulfonamides (SA), tetracyclines (TC), neomycin (NEO), tobramycin (TOB), kanamycin (KAN), ampicillin (AMP).

Targets	Bioreceptors	LoD	Transduction & Analytical Methods	Ref.
CF	Ab	3 nM	EIS	[5]
CF	Ab	30 pM	EIS	[6]
CF	Ab	3 nM	EIS	[7]
CF	Ab	3 nM	SAM/EIS	[8]
OFL	Ab	0.1 nM	HRP/AuNPs/PPy/CV	[9]
SA	Ab	6 nM	MBs/HRP/CV	[10]
TC	Ab	4 nM	MBs/HRP/CV	[11]
TC	Ab	1 nM	HRP/HQ/CV	[12]
TC	Ab	13 pM	Platinum-catalyzed HER/CV	[14]
TC	Aptamer	1 nM	Fe(CN)_6_^3−/4−^/CV	[15]
TC	Aptamer	10 nM	Fe(CN)_6_^3−/4−^/CV/SWV	[16]
TC	Aptamer	5 pM	CNTs/Fe(CN)_6_^3−/4−^/DPV	[17]
TC	Aptamer	2 nM	EIS	[18]
TC	Aptamer	0.3 nM	PB/DPV	[19]
NEO	Aptamer	1 μM	EIS	[20]
TOB	Aptamer	2 μM	MB label/CV	[21]
TOB	Aptamer	1 mM	AuNPs/MB label/CV	[22]
TOB	Aptamer	5 μM	MBs/AlkP/CV	[23]
TOB	Aptamer	0.1 μM	MBs/AlkP/CV	[24]
KAN	Aptamer	10 nM	AuNPs/CV	[25]
KAN AMP	Aptamer Aptamer	10 nM 100 pM	PEDOT/EIS PEDOT/EIS	[26] [26]

Note: LoD (limit of detection); HRP (horseradish peroxidase); PPy (polypyrrole); HER (hydrogen evolution reaction), CV (cyclic voltammetry); SWV (square wave voltammetry); MBs (magnetic beads); CNTs (carbon nanotubes); PB (prussian blue); MB (methylene blue); AuNPs (gold nanoparticles).

**Table 2:** Figures of merit of selected immunosensors, aptasensors and peptide sensors for detection of bisphenol A.

Bioreceptors	LoD	Transduction and Analytical Methods	Ref.
Ab	1.3 nM	Conducting polymer & EIS	[28,29]
Ab	9 pM	Conducting polymer & SWV	[30]
Aptamer	1 pM	Fe(CN)_6_^3−/4^/EIS	[31]
Aptamer	5 nM	Graphene/Fe(CN)_6_^3−/4^/DPV	[32]
Peptide	1 nM	SAM/Fe(CN)_6_^3−/4^/DPV	[33]
Peptide	100 fM	Graphene oxide/EIS	[34]

**Table 3:** Figures of merit of selected immunosensors and aptasensors for detection of cocaine.

Bioreceptors	LoD	Transduction and Analytical Methods	Ref.
Ab	380 pM	AlkP-Ab/CV	[35]
Ab	0.1 μM	HRP-Ab/CV	[36]
Ab	33 fM	SAM/EIS	[37]
Aptamer	10 μM	MB-labeled/CV	[39]
Aptamer	1 μM	Two-strand aptamer/SWV	[40]
Aptamer	1 μM	PtNPs-aptamer/H_2_O_2_/CV	[41]
Aptamer	0.1 μM	Label-free/Two-strand aptamer/CV	[42]
Aptamer	50 nM	Two-strand aptamer/QDs	[43]
Aptamer	1 nM	Two-strand aptamer/QDs	[44]
Aptamer	100 nM	Two-strand aptamer/EIS/[Fe(CN)_6_]^3−/4−^	[45]
Aptamer	100 pM	Two-strand aptamer/AuNPs/DPV/[Fe(CN)_6_]^3−/4−^	[46]
Aptamer	150 pM	Two-strand aptamer/AgNPs/CV/riboflavin	[47]
Aptamer	1 nM	Two-strand aptamer/AlkP/CV	[48]
Aptamer	20 nM	Two-strand aptamer/HRP/CV	[49]
Aptamer	1 nM	RCA/AlkP/DPV	[50]

**Table 4:** Figures of merit of selected immunosensors and aptasensors for ochratoxin A.

Bioreceptors	LoD	Transduction and Analytical Methods	Reference
Ab	0.8 nM	AlkP- or HRP-Ab/CV	[51]
Ab	30 nM	HRP/TMB/chronoamperometry	[52]
Ab	1.3 nM	HRP/TMB/EIS	[53]
Ab	25 pM	MBs/EIS	[54]
Ab	0.12 nM	HRP/TMB/EIS	[55]
Ab	17 pM	BDD/EIS	[56]
Ab	5 pM	CdSe/TiO_2_/PEC	[57]
Aptamer	80 pM	Multiple stem/MB-DNA/CV	[58]
Aptamer	0.25 nM	LB/PANI/EIS	[59]
Aptamer	2.5 pM	Fc-labeled aptamer/exonuclease/CV	[60]
Aptamer	1 pM	HRP-labeled aptamer/exonuclease/CV	[61]
Aptamer	0.3 pM	Fe(CN)_6_^3−/4−^/EIS	[62]

Note: TMB (3,3′,5,5′-tetramethylbenzidine); PEC (photoelectrochemical); BDD (boron doped diamond); MB (methylene blue); LB (Langmuir Blodgett); PANI (polyaniline).

**Table 5:** Figures of merit of selected immunosensors and aptasensors for detection of estradiol.

Bioreceptors	LoD	Transduction and Analytical Methods	Reference
Ab	0.1 nM	SAM/BQ/CV	[67]
Ab	0.2 nM	AlkP-estradiol/DPV	[68]
Ab	0.9 pM	AlkP-estradiol/amperometry	[69]
Ab	0.15 nM	AlkP-estradiol/amperometry	[70]
Ab	20 pM	AuNPs/HRP/BQ/CV	[71]
Ab	65 pM	AuNPs/Protein G/[Fe(CN)_6_]^3−/4−^/SWV	[72]
Ab	95 pM	AuNPs/Protein G/[Fe(CN)_6_]^3−/4−^/EIS	[72]
Ab	1 pM	MBs/HRP-estradiol	[73]
Ab	0.1 pM	HRP-estradiol/Catechol/CV	[74]
Ab	45 pM	Protein G/Fc-MeOH/SWV	[75]
Ab	2.8 pM	PABA/BQ	[76]
Ab	3.6 pM	MBs/AlkP-αIgG/SWV	[77]
Ab	0.2 nM	CdSe QDs/dissolution (SWASV)	[78]
Ab	75 pM	Graphene/HRP-GO	[79]
Ab	28 pM	Cu_2_S NPs/CV	[80]
Ab	55 fM	Pb^2+^, Cd^2+^/porous Fe_3_O_4_/SWASV	[81]
Ab	3 pM	AuNPs	[82]
Aptamer	0.1 nM	PEDOT/AuNPs/Biot-Avidin/[Fe(CN)_6_]^3−/4−^/CV-SWV	[83]
Aptamer	2 pM	SAM/Au/[Fe(CN)_6_]^3−/4−^/EIS	[84]
Aptamer	1 pM	VS_2_/[Fe(CN)_6_]^3−/4−^/DPV	[85]
Aptamer	5 fM	Au/BDD/[Fe(CN)_6_]^3−/4−^/EIS	[86]
Aptamer	1 fM	Nanoporous electrode/[Fe(CN)_6_]^3−/4−^/EIS	[87]
Aptamer	1 pM	Ni^3+^,Fe(CN)_6_]^3−^/AuNPs	[88]
Aptamer	3 nM	SAM/Au/[Fe(CN)_6_]^3−/4−^/EIS	[89]

Note: BQ (benzoquinone); Fc-MeOH (ferrocenemethanol); PABA (p-aminobenzoic acid); SWASV (square wave adsorptive stripping voltammetry).

## References

[1] Sarma V.R., Silverton E.W., Davies D.R., Terry W.D. (1971). The three-dimensional structure at 6 A resolution of a human gamma Gl immunoglobulin molecule. J. Biol. Chem..

[2] Silverton E.W., Navia M.A., Davies D.R. (1977). Three-dimensional structure of an intact human immunoglobulin. Proc. Natl. Acad. Sci. USA.

[3] Antibodies-Online. http://xww.antibodies-online.com.

[4] Mascini M., Palchetti I., Tombelli S. (2012). Nucleic Acid and Peptide Aptamers: Fundamentals and Bioanalytical Aspects. Angew. Chem. Int. Ed..

[5] Garifallou G.Z., Tsekenis G., Davis F., Higson S.P.J., Millner P.A., Pinacho D.G., Sanchez-Baeza F., Marco M.P., Gibson T.D. (2007). Labeless immunosensor assay for fluoroquinolone antibiotics based upon an AC impedance protocol. Anal. Lett..

[6] Ionescu R.E., Jaffrezic-Renault N., Bouffier L., Gondran C., Cosnier S., Pinacho D.G., Marco M.P., Sanchez-Baeza F.J., Healy T., Martelet C. (2007). Impedimetric immunosensor for the specific label free detection of ciprofloxacin antibiotic. Biosens. Bioelectron..

[7] Giroud F., Gorgy K., Gondran C., Cosnier S., Pinacho D.G., Marco M.P., Sánchez-Baeza F.J. (2009). Impedimetric immunosensor based on a polypyrrole-antibiotic model film for the label-free picomolar detection of ciprofloxacin. Anal. Chem..

[8] Wu C.C., Lin C.H., Wang W.S. (2009). Development of an enrofloxacin immunosensor based on label-free electrochemical impedance spectroscopy. Talanta.

[9] Zang S., Liu Y., Lin M., Kang J., Sun Y., Lei H. (2013). A dual amplified electrochemical immunosensor for ofloxacin: Polypyrrole film-Au nanocluster as the matrix and multi-enzyme-antibody functionalized gold nanorod as the label. Electrochim. Acta.

[10] Zacco E., Adrian J., Galve R., Marco M.P., Alegret S., Pividori M.I. (2007). Electrochemical magneto immunosensing of antibiotic residues in milk. Biosens. Bioelectron..

[11] Centi S., Stoica A.I., Laschi S., Mascini M. (2010). Development of an Electrochemical Immunoassay Based on the Use of an Eight-Electrodes Screen-Printed Array Coupled with Magnetic Beads for the Detection of Antimicrobial Sulfonamides in Honey. Electroanalysis.

[12] Conzuelo A.F., Gamella M., Campuzano S., Reviejo A.J., Pingarrón J.M. (2012). Disposable amperometric magneto-immunosensor for direct detection of tetracyclines antibiotics residues in milk. Anal. Chim. Acta.

[13] Conzuelo F., Campuzano S., Gamella M., Pinacho D.G., Reviejo A.J., Marco M.P., Pingarrón J.M. (2013). Integrated disposable electrochemical immunosensors for the simultaneous determination of sulfonamide and tetracycline antibiotics residues in milk. Biosens. Bioelectron..

[14] Que X., Chen X., Fu L., Lai W., Zhuang J., Chen G., Tang D. (2013). Platinum-catalyzed hydrogen evolution reaction for sensitive electrochemical immunoassay of tetracycline residues. J. Electroanal. Chem..

[15] Kim Y.S., Niazi J.H., Gu M.B. (2009). Specific detection of oxytetracycline using DNA aptamer-immobilized interdigitated array electrode chip. Anal. Chim. Acta.

[16] Kim Y.J., Kim Y.S., Niazi J.H., Gu M.G. (2010). Electrochemical aptasensor for tetracycline detection. Bioprocess. Biosyst. Eng..

[17] Zhou L., Li D.J., Gai L., Wang J.P., Li Y.B. (2012). Electrochemical aptasensor for the detection of tetracycline with multi-walled carbon nanotubes amplification. Sens. Actuators B Chem..

[18] Chen D., Yao D., Xie C., Liu D. (2014). Development of an aptasensor for electrochemical detection of tetracycline. Food Control.

[19] Shen G., Guo Y., Sun X., Wang X. (2014). Electrochemical Aptasensor Based on Prussian Blue-Chitosan-Glutaraldehyde for the Sensitive Determination of Tetracycline. Nano-Micro Lett..

[20] De-los-Santos-Álvarez N., Lobo-Castañón M.J., Miranda-Ordieres A.J., Tuñón-Blanco P. (2007). Modified-RNA aptamer-based sensor for competitive impedimetric assay of neomycin B. J. Am. Chem. Soc..

[21] Rowe A.A., Miller E.A., Plaxco K.W. (2010). Reagentless Measurement of Aminoglycoside Antibiotics in Blood Serum via an Electrochemical, Ribonucleic Acid Aptamer-Based Biosensor. Anal. Chem..

[22] Liu J., Wagan S., Morris M.D., Taylor J., White R.J. (2014). Achieving Reproducible Performance of Electrochemical, Folding Aptamer-Based Sensors on Microelectrodes: Challenges and Prospects. Anal. Chem..

[23] González-Fernández E., de-los-Santos-Álvarez N., Lobo-Castañón M.J., Miranda-Ordieres A.J., Tuñón-Blanco P. (2011). Aptamer-Based Inhibition Assay for the Electrochemical Detection of Tobramycin Using Magnetic Microparticles. Electroanalysis.

[24] González-Fernández E., de-los-Santos-Álvarez N., Miranda-Ordieres A.J., Lobo-Castañón M.J. (2013). Monovalent labeling system improves the sensitivity of aptamer-based inhibition assays for small molecule detection. Sens. Actuators B Chem..

[25] Zhu Y., Chandra P., Song K.M., Ban C., Shim Y.B. (2012). Label-free detection of kanamycin based on the aptamer-functionalized conducting polymer/gold nanocomposite. Biosens. Bioelectron..

[26] Daprà J., Lauridsen L.H., Nielsen A.T., Rozlosnik N. (2013). Comparative study on aptamers as recognition elements for antibiotics in a label-free all-polymer biosensor. Biosens. Bioelectron..

[27] Ragavan K.V., Rastogi N.K., Thakur M.S. (2013). Sensors and biosensors for analysis of bisphenol-A. TRAC Trend Anal. Chem..

[28] Rahman M.A., Shiddiky M.J.A., Park J.S., Shim Y.B. (2007). An impedimetric immunosensor for the label-free detection of bisphenol A. Biosens. Bioelectron..

[29] Piao M.H., Noh H.B., Rahman M.A., Won M.S., Shim Y.B. (2008). Label-Free Detection of Bisphenol A Using a Potentiometric Immunosensor. Electroanalysis.

[30] Wang X., Reisberg S., Serradji N., Anquetin G., Pham M.C., Wu W., Dong C.Z., Piro B. (2014). E-assay concept: Detection of bisphenol A with a label-free electrochemical competitive immunoassay. Biosens. Bioelectron..

[31] Xue F., Wu J.J., Chu H.Q., Mei Z.L., Ye Y.K., Liu J., Zhang R., Peng C.F., Zheng L., Chen W. (2013). Electrochemical aptasensor for the determination of bisphenol A in drinking water. Microchim. Acta.

[32] Zhou L., Wang J., Li D., Li Y. (2014). An electrochemical aptasensor based on gold nanoparticles dotted graphene modified glassy carbon electrode for label-free detection of bisphenol A in milk samples. Food Chem..

[33] Yang J., Kim S.E., Cho M., Yoo I.K., Choe W.S., Lee Y. (2014). Highly sensitive and selective determination of bisphenol-A using peptide-modified gold electrode. Biosens. Bioelectron..

[34] Kim K.S., Jang J.R., Choe W.S., Yoo P.J. (2015). Electrochemical detection of Bisphenol A with high sensitivity and selectivity using recombinant protein-immobilized graphene electrodes. Biosens. Bioelectron..

[35] Bauer C.G., Eremenko A.V., Kühn A., Kürzinger K., Makower A., Scheller F.W. (1998). Anal. Chem..

[36] Suleiman A.A., Xu Y.H. (1998). An amperometric immunosensor for cocaine. Electroanalysis.

[37] Yang Y., Pan J.Y., Hua W.J., Tu Y.F. (2014). An approach for the preparation of highly sensitive electrochemical impedimetric immunosensors for the detection of illicit drugs. J. Electroanal. Chem..

[38] Ozkan S.A., Kauffmann J.M., Zuman P. (2015). Electrochemical Biosensors for Drug Analysis. Electroanalysis in Biomedical and Pharmaceutical Sciences.

[39] Baker B.R., Lai R.Y., Wood M.S., Doctor E.H., Heeger A.J., Plaxco K.W. (2006). An Electronic, Aptamer-Based Small-Molecule Sensor for the Rapid, Label-Free Detection of Cocaine in Adulterated Samples and Biological Fluids. J. Am. Chem. Soc..

[40] Zuo X., Xiao Y., Plaxco K.W. (2009). High Specificity, Electrochemical Sandwich Assays Based on Single Aptamer Sequences and Suitable for the Direct Detection of Small-Molecule Targets in Blood and Other Complex Matrices. J. Am. Chem. Soc..

[41] Golub E., Pelossof G., Freeman R., Zhang H., Willner I. (2009). Electrochemical, Photoelectrochemical, and Surface Plasmon Resonance Detection of Cocaine Using Supramolecular Aptamer Complexes and Metallic or Semiconductor Nanoparticles. Anal. Chem..

[42] Du Y., Chen C., Yin J., Li B., Zhou M., Dong S., Wang E. (2010). Solid-State Probe Based Electrochemical Aptasensor for Cocaine: A Potentially Convenient, Sensitive, Repeatable, and Integrated Sensing Platform for Drugs. Anal. Chem..

[43] Zhang H., Jiang B., Xiang Y., Zhang Y., Chai Y., Yuan R. (2011). Aptamer/quantum dot-based simultaneous electrochemical detection of multiple small molecules. Anal. Chim. Acta.

[44] Hua M., Li P., Li L., Huang L., Zhao X., Feng Y., Yang Y. (2011). Quantum dots as immobilized substrate for electrochemical detection of cocaine based on conformational switching of aptamer. J. Electroanal. Chem..

[45] Zhang D.W., Zhang F.T., Cui Y.R., Deng Q.P., Krause S., Zhou Y.L., Zhang X.X. (2012). A label-free aptasensor for the sensitive and specific detection of cocaine using supramolecular aptamer fragments/target complex by electrochemical impedance spectroscopy. Talanta.

[46] Roushani M., Shahdost-fard F. (2015). A highly selective and sensitive cocaine aptasensor based on covalent attachment of the aptamer-functionalized AuNPs onto nanocomposite as the support platform. Anal. Chim. Acta.

[47] Roushani M., Shahdost-fard F. (2015). A novel ultrasensitive aptasensor based on silver nanoparticles measured via enhanced voltammetric response of electrochemical reduction of riboflavin as redox probe for cocaine detection. Sens. Actuators B Chem..

[48] Jiang B., Wang M., Chen Y., Xie J., Xiang Y. (2012). Highly sensitive electrochemical detection of cocaine on graphene/AuNP modified electrode via catalytic redox-recycling amplification. Biosens. Bioelectron..

[49] Zhang D.W., Sun C.J., Zhang F.T., Xu L., Zhou Y.L., Zhang X.X. (2012). An electrochemical aptasensor based on enzyme linked aptamer assay. Biosens. Bioelectron..

[50] Shen B., Li J., Cheng W., Yan Y., Tang R., Li Y., Ju H., Ding S. (2015). Electrochemical aptasensor for highly sensitive determination of cocaine using a supramolecular aptamer and rolling circle amplification. Microchim. Acta.

[51] Prieto-Simon B., Campas M., Marty J.L., Noguer T. (2008). Novel highly-performing immunosensor-based strategy for ochratoxin A detection in wine samples. Biosens. Bioelectron..

[52] Radi A.E., Munoz-Berbel X., Cortina-Puig M., Marty J.L. (2009). An electrochemical immunosensor for ochratoxin A based on immobilization of antibodies on diazonium-functionalized gold electrode. Electrochim. Acta.

[53] Radi A.E., Munoz-Berbel X., Lates V., Marty J.L. (2009). Label-free impedimetric immunosensor for sensitive detection of ochratoxin A. Biosens. Bioelectron..

[54] Zamfir L.G., Geana I., Bourigua S., Rotariu L., Bala C., Errachid A., Jaffrezic-Renault N. (2011). Highly sensitive label-free immunosensor for ochratoxin A based on functionalized magnetic nanoparticles and EIS/SPR detection. Sens. Actuators B Chem..

[55] Heurich M., Abdul Kadir M.K., Tothill I.E. (2011). An electrochemical sensor based on carboxymethylated dextran modified gold surface for ochratoxin A analysis. Sens. Actuators B Chem..

[56] Chrouda A., Sbartai A., Bessueille F., Renaud L., Maaref A., Jaffrezic-Renault N. (2015). Electrically addressable deposition of diazonium functionalized antibodies on boron-doped diamond microcells for the detection of ochratoxin A. Anal. Methods.

[57] Yang J., Gao P., Liu Y., Li R., Ma H., Du B., Wei Q. (2015). Label-free photoelectrochemical immunosensor for sensitive detection of Ochratoxin A. Biosens. Bioelectron..

[58] Kuang H., Chen W., Xu D., Xu L., Zhu Y., Liu L., Chu H., Peng C., Xu C., Zhu S. (2010). Fabricated aptamer-based electrochemical “signal-off” sensor of ochratoxin A. Biosens. Bioelectron..

[59] Prabhakar N., Matharu Z., Malhotra B.D. (2011). Polyaniline Langmuir–Blodgett film based aptasensor for ochratoxin A detection. Biosens. Bioelectron..

[60] Tong P., Zhang L., Xu J.J., Chen H.Y. (2011). Simply amplified electrochemical aptasensor of Ochratoxin A based on exonuclease-catalyzed target recycling. Biosens. Bioelectron..

[61] Zhang J., Chen J., Zhang X., Zeng Z., Chen M., Wang S. (2012). An electrochemical biosensor based on hairpin-DNA aptamer probe and restriction endonuclease for ochratoxin A detection. Electrochem. Commun..

[62] Hayat A., Andreescu S., Marty J.L. (2013). Design of PEG-aptamer two piece macromolecules as convenient and integrated sensing platform: Application to the label-free detection of small size molecules. Biosens. Bioelectron..

[63] Bazin I., Andreotti N., Hassine A.I., De Waard M., Sabatier J.M., Gonzalez C. (2013). Peptide binding to ochratoxin A mycotoxin: A new approach in conception of biosensors. Biosens. Bioelectron..

[64] Heurich M., Altintas Z., Tothill I.E. (2013). Computational Design of Peptide Ligands for Ochratoxin A. Toxins.

[65] Soleri R., Demey H., Tria S.A., Guiseppi-Elie A., Hassine A.I., Gonzalez C., Bazin I. (2015). Peptide conjugated chitosan foam as a novel approach for capture-purification and rapid detection of hapten—Example of ochratoxin A. Biosens. Bioelectron..

[66] Padeste C., Grubelnik A., Tiefenauer L. (1998). Amperometric immunosensing using microperoxidase MP-11 antibody conjugates. Anal. Chim. Acta.

[67] Kuramitz H., Matsuda M., Thomas J.H., Sugawara K., Tanaka S. (2003). Electrochemical immunoassay at a 17 beta-estradiol self-assembled monolayer electrode using a redox marker. Analyst.

[68] Pemberton R.M., Mottram T.T., Hart J.P. (2005). Development of a screen-printed carbon electrochemical immunosensor for picomolar concentrations of estradiol in human serum extracts. J. Biochem. Biophys. Methods.

[69] Butler D., Guilbault G.G. (2006). Disposable amperometric immunosensor for the detection of 17-beta estradiol using screen-printed electrodes. Sens. Actuators B Chem..

[70] Volpe G., Fares G., Quadri F., delli Draisci R., Ferretti G., Marchiafava C., Moscone D., Palleschi G. (2006). A disposable immunosensor for detection of 17beta-estradiol in non-extracted bovine serum. Anal. Chim. Acta.

[71] Liu X., Wong D.K.Y. (2009). Picogram-detection of estradiol at an electrochemical immunosensor with a gold nanoparticle vertical bar Protein G-(LC-SPDP)-scaffold. Talanta.

[72] Liu X., Duckworth P.A., Wong D.K.Y. (2010). Square wave voltammetry *versus* electrochemical impedance spectroscopy as a rapid detection technique at electrochemical immunosensors. Biosens. Bioelectron..

[73] Martinez N.A., Schneider R.J., Messina G.A., Raba J. (2010). Modified paramagnetic beads in a microfluidic system for the determination of ethinylestradiol (EE2) in river water samples. Biosens. Bioelectron..

[74] Kim B.K., Li J., Im J.E., Ahn K.S., Park T.S., Cho S.I., Kim Y.R., Lee W.Y. (2012). Impedometric estrogen biosensor based on estrogen receptor alpha-immobilized gold electrode. J. Electroanal. Chem..

[75] Liu X., Wang X., Zhang J., Feng H., Liu X., Wong D.K.Y. (2012). Detection of estradiol at an electrochemical immunosensor with a Cu UPD vertical bar DTBP-Protein G scaffold. Biosens. Bioelectron..

[76] Ojeda I., Lopez-Montero J., Moreno-Guzman M., Janegitz B.C., Gonzalez-Cortes A., Yanez-Sedeno P., Pingarron J.M. (2012). Electrochemical immunosensor for rapid and sensitive determination of estradiol. Anal. Chim. Acta.

[77] Kanso H., Barthelmebs L., Inguimbert N., Noguer T. (2013). Immunosensors for Estradiol and Ethinylestradiol Based on New Synthetic Estrogen Derivatives: Application to Wastewater Analysis. Anal. Chem..

[78] Chaisuwan N., Xu H., Wu G., Liu J. (2013). A highly sensitive differential pulse anodic stripping voltammetry for determination of 17 beta-estradiol (E2) using CdSe quantum dots based on indirect competitive immunoassay. Biosens. Bioelectron..

[79] Li J., Liu S., Yu J., Lian W., Cui M., Xu W., Huang J. (2013). Electrochemical immunosensor based on graphene-polyaniline composites and carboxylated graphene oxide for estradiol detection. Sens. Actuators B Chem..

[80] Zhang S., Wang Y., Zhang Y., Yan T., Yan L., Wei Q., Du B. (2015). An ultrasensitive electrochemical immunosensor for determination of estradiol using coralloid Cu_2_S nanostructures as labels. RSC Adv..

[81] Zhang S., Du B., Li H., Xin X., Ma H., Wu D., Yan L., Wei Q. (2014). Metal ions-based immunosensor for simultaneous determination of estradiol and diethylstilbestrol. Biosens. Bioelectron..

[82] Monerris M.J., Arévalo F.J., Fernández H., Zon M.A., Molina P.G. (2015). Development of a very sensitive electrochemical immunosensor for the determination of 17β-estradiol in bovine serum samples. Sens. Actuators B Chem..

[83] Olowu R.A., Arotiba O., Mailu S.N., Waryo T.T., Baker P., Iwuoha E. (2010). Electrochemical Aptasensor for Endocrine Disrupting 17 beta-Estradiol Based on a Poly(3,4-ethylenedioxylthiopene)-Gold Nanocomposite Platform. Sensors.

[84] Lin Z., Chen L., Zhang G., Liu Q., Qiu B., Cai Z., Chen G. (2012). Label-free aptamer-based electrochemical impedance biosensor for 17 beta-estradiol. Analyst.

[85] Huang K.J., Liu Y.J., Shi G.W., Yang X.R., Liu Y.M. (2014). Label-free aptamer sensor for 17 beta-estradiol based on vanadium disulfide nanoflowers and Au nanoparticles. Sens. Actuators B Chem..

[86] Ke H., Liu M., Zhuang L., Li Z., Fan L., Zhao G. (2014). A Fetomolar Level 17 beta-estradiol Electrochemical Aptasensor Constructed On Hierachical Dendritic Gold Modified Boron-Doped Diamond Electrode. Electrochim. Acta.

[87] Zhu B., Alsager O.A., Kumar S., Hodgkiss J.M., Travas-Sejdic J. (2015). Label-free electrochemical aptasensor for femtomolar detection of 17 beta-estradiol. Biosens. Bioelectron..

[88] Fan L., Zhao G., Shi H., Liu M. (2015). A simple and label-free aptasensor based on nickel hexacyanoferrate nanoparticles as signal probe for highly sensitive detection of 17β-estradiol. Biosens. Bioelectron..

[89] Jimenez G.C., Eissa S., Ng A. (2015). Aptamer-Based Label-Free Impedimetric Biosensor for Detection of Progesterone. Anal. Chem..

